# An Overview of Bio-Based Polymers with Potential for Food Packaging Applications

**DOI:** 10.3390/polym17172335

**Published:** 2025-08-28

**Authors:** Raluca Şomoghi, Sonia Mihai, Florin Oancea

**Affiliations:** 1 Faculty of Petroleum Refining and Petrochemistry, Petroleum-Gas University of Ploiesti, 100680 Ploiesti, Romania; smihai@upg-ploiesti.ro; 2 National Institute for Research and Development in Chemistry and Petrochemistry—ICECHIM, 060021 Bucharest, Romania; florino@ping.ro

**Keywords:** bio-based polymers, food packaging, mechanical properties, barrier qualities, biodegradability, sustainability

## Abstract

Food packaging is an essential part of the food industry. Packaging materials are indispensable in ensuring product safety, enhancing consumer experience, and supporting sustainable practices. This review provides an update on the role of bio-based polymers, including polylactic acid (PLA), polyhydroxyalkanoates (PHAs), starch-based polymers, and cellulose-based polymers (cellulose acetate (CA), cellulose sulphate (CS), carboxymethyl cellulose (CMC), nanocellulose (NC), and methylcellulose (MC)) for food packaging applications. Properties as mechanical, barrier and antimicrobial, as well as their eco-friendly behavior, are also summarized. The advantages and disadvantages of using bio-based polymers in food packaging are discussed. Present review also addresses the challenges associated with their preparation and highlights the potential future prospects of bio-based polymers for packaging applications.

## 1. Introduction

As global concern over environmental sustainability continues to grow, the search for alternative materials to replace petrochemical plastics has intensified. To assess the health impacts of food packaging, it is essential to understand the chemical composition and physical characteristics of the packaging material [1,2,3].

The packaging industry is undergoing rapid transformation, determined by sustainability goals and consumer preferences. Some notable trends include: (i) eco-friendly packaging (a shift toward recyclable, compostable, or reusable materials); (ii) minimalist design (clean, simple packaging that reduces waste and appeals to modern aesthetics); (iii) smart packaging (integration of sensors to enhance product interaction and traceability); and (iv) customization (personalized packaging solutions for targeted marketing and better customer engagement) [4,5].

Food packaging, which has long relied on petroleum-based polymers, is a sector where the shift to more sustainable materials could have a significant impact in the modern food industries. They perform many important functions, from protecting food to facilitating transportation, storage and sale. With increasing awareness of health, hygiene and sustainability, the role of food packaging is becoming more important [6,7]. Food can be packaged using a wide range of materials, each chosen based on the type of food and how it will be used, such as plastic (utilized for bottles, containers, films, and wraps) [8,9], glass (ideal for liquids like sauces and drinks) [9], metal (often used for canned foods and beverages) [10], paper and cardboard (common in dry food packaging like cereal boxes, bakery items, and fast food) [11], and biodegradable materials made from natural sources (e.g., cornstarch or sugarcane) [12]. While food packaging plays a vital role, it also contributes to pollution, especially plastic waste. Many packaging materials are single-use and end up in landfills or oceans. As a result, there is a growing push toward recycling and using recycled materials, reducing the amount of packaging used, replacing plastics with biodegradable or compostable alternatives, encouraging consumers to choose reusable or eco-friendly packaging.

**Figure 1:**
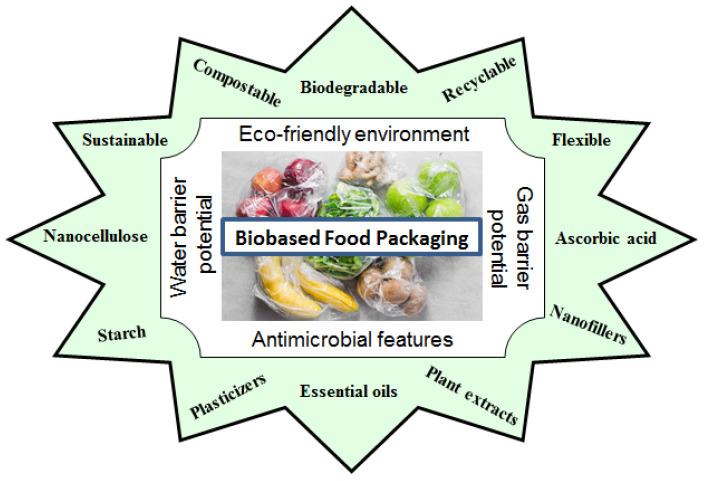
General properties of bio food packaging materials.

Bio-based polymers are polymeric materials that represent a crucial step towards a more sustainable future because they are derived from renewable biodegradable resources (e.g., plants, algae, or microorganisms). Unlike, conventional plastics which are made from fossil fuels, bio-based polymers are sourced from materials, such as corn starch, sugarcane, cellulose, or vegetable oils. They offer a potential way to reduce the environmental footprint of food packaging while maintaining functionality, quality, safety, sustainability, and cost-effectiveness [13,14]. In the food packaging industry, bio-based polymers are being investigated for their potential to replace traditional plastics, including polyethylene (PE), polypropylene (PP), polystyrene (PS), and polyamides, which are produced only by synthetic methods and contribute significantly to global plastic pollution. Several bio-based polymers (e.g., polylactic acid (PLA), polyhydroxyalkanoates (PHAs), starch, and cellulose) are currently used or under development for sustainable packaging solutions [15]. These biopolymers present some advantages in food packaging, such as: (1) are eco-friendly and sustainable (biodegradable and reduce plastic waste in the environment), (2) are derived from renewable resources (plant-based or microbial sources), (3) are compostable (can be degraded in composting facilities, reducing the use of landfills), and (4) offer various functional benefits (antimicrobial or oxygen barrier properties) that extend the shelf life of food when enriched with essential oils, plant extracts, or nanoparticles [16]. By utilizing plant-based feedstocks, the food packaging industry can help create a more circular economy [17,18]. Bio-based polymers can form coatings and films that block foodborne pathogens and limit gas transmission. Some bio-based materials, such as PLA films, cellulose-based films, can be mixed with plasticizers or other polymers to enhance their flexibility and durability (Figure 1) [19]. However, they have limitations in mechanical strength and moisture resistance, which can hinder their practical application [20].

Despite numerous papers reporting the use of bio-based polymers for packaging [21,22,23,24], the novelty of this review lies in explaining the role of bio-based polymers, highlighting the specific types of these polymers, such as polylactic acid (PLA), polyhydroxyalkanoates (PHAs), starch-based polymers, and cellulose-based polymers (cellulose acetate (CA), cellulose sulphate (CS), carboxymethyl cellulose (CMC), nanocellulose (NC), and methylcellulose (MC)), suitable for different food packaging. It also examines the current state of research, industrial applications, and the advantages and disadvantages of using bio-based polymers in food packaging. Mechanical, barrier, and antimicrobial properties, as well as their eco-friendly behavior of bio-based polymers are included to present the selection criteria for materials that can be used in food service applications. The relationship between biopolymers compositions and their properties is presented to obtain bio-based materials with specific characteristics for targeted applications. Furthermore, the review addresses the challenges associated with wider adoption of bio-based packaging and suggests future research directions for making it more affordable and accessible.

## 2. Bio-Based Polymers for Food Packaging

### 2.1. Polylactic Acid (PLA)

PLA is one of the most well-known bio-based polymers. This biopolymer has attracted significant attention in the food packaging industry, as it is a biodegradable thermoplastic aliphatic polyester, derived from fermented vegetable starch (corn or sugarcane), being a renewable alternative to petroleum-based plastics [25]. Its biodegradability requires specific environmental conditions, such as heat and moisture, which may not always be available in natural environments, limiting its effectiveness as a solution for end-of-life disposal.

**Figure 2:**
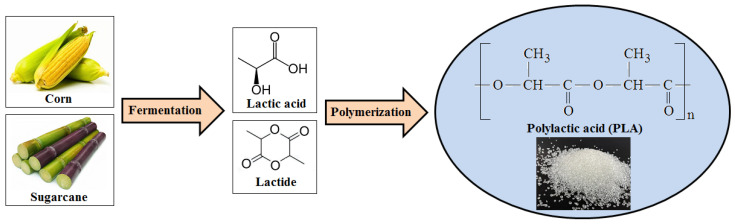
Schematic representation for production of PLA used as bio-based polymer.

PLA is synthesized starting from fermentation of renewable resources (e.g., corn or sugarcane) to produce lactic acid (LA) followed by polymerization. The formation of lactide often serves as an intermediate step (Figure 2). Various polymerization methods, such as direct polycondensation, enzymatic polymerization, azeotropic dehydration, and ring-opening polymerization, can be employed to synthesize PLA from LA [26,27,28].

The role of PLA in food packaging is to reduce environmental impact. PLA helps reduce plastic pollution and dependence on fossil fuels because it is made from plants and decomposes more easily than regular plastic. This biopolymer is non-toxic and food-safe, making it a good choice for packaging fresh, vegetables, baked goods, and takeaway food. It does not release harmful chemicals into the food. Tone et al. [29] created an active food packaging material using PLA as the base matrix, incorporating a bioactive compound derived from orange peel. The resulting transparent film can be used for packaging applications of fresh products sensitive to oxidation. Moraczewski et al. [30] used a blend of polylactic acid (PLA)/polycaprolactone (PCL) polymers and tannic acid as an antimicrobial agent to develop an active material suitable for food packaging applications. The results indicated that the PLA/PCL blend with 5 wt% tannic acid can be considered a promising candidate for use in active food packaging materials, providing an effective balance of antimicrobial performance, consumer safety, and environmental sustainability.

### 2.2. Polyhydroxyalkanoates (PHAs)

**Figure 3:**
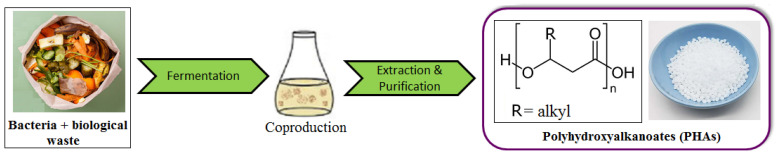
Schematic representation for production of PHAs used as bio-based polymer.

PHAs are a group of bio-based polymers produced by bacteria through the fermentation of renewable resources, such as sugar, starch, or vegetable oils, under nutrient-limited conditions (e.g., nitrogen, phosphorus, or oxygen limitation) with an excess supply of carbon [31]. PHAs are composed of (R)-hydroxyalkanoate (HA) units, arranged into a basic structure obtained by bacterial fermentation (e.g., *Cupriavidus necator*, or *Psudomonas putida*). Once fermentation is complete, PHAs can be extracted from the bacterial cells, using methods such as solvent extraction, and mechanical disruption followed by purification (see Figure 3) [32]. The purity and molecular weight of the extracted PHA influence its mechanical properties, which are critical for food packaging applications. It has been reported that the diversity of PHAs comes from the structural variation of their monomeric units, especially in the alkyl side chains (R) [33]. Literature data have shown that PHAs can be synthesized by a wide range of bacteria (e.g., *Ralstonia eutropha*, *Alcaligenes latus*, *Haloferax mediterranei* and *Bacillus*) under specific conditions, usually when a carbon source is in excess and other essential nutrients, such as nitrogen, phosphorus, or oxygen, are limited. These stressful conditions trigger the microorganisms to store carbon in the form of PHAs [34,35]. Unlike PLA, PHAs are biodegradable under various environmental conditions, including soil, marine, and industrial composting environments [36]. They offer a potential solution to the growing problem of marine plastic pollution. However, their production costs remain high, which limits their widespread adoption.

The two most studied types of PHA are poly(3-hydroxybutyrate) (PHB), which is the most common PHA, and poly(3-hydroxybutyrate-co-3-hydroxyvalerate) (PHBV). PHB is a linear polyester composed of repeating units of 3-hydroxybutyrate and is naturally produced by a wide range of bacteria, including *Cupriavidus necator*, *Alcaligenes latus*, *Ralstonia eutropha* and *Bacillus megaterium* [37]. PHBV is a copolymer of 3-hydroxybutyrate (3HB) and 3-hydroxyvalerate (3HV), both of which are naturally produced by microorganisms (e.g., *Cupriavidus necator*, *Azotobacter vinelandii*, and *Pseudomonas oleovorans*) through fermentation [38]. PHB exhibits poor flexibility, low impact strength, and high stiffness, which are major concerns for packaging applications that require durability and strength. The incorporation of 3HV units into the polymer chain reduces the brittleness of PHB and increases its flexibility and processability. Combining PHB with more flexible biopolymers (e.g., polycarpolactone or PLA) can improve toughness.

The key role of PHAs in food packaging is their ability to naturally decompose in both industrial and home composting environments, as well as in marine environments. This property makes them ideal for single-use food packaging, such as containers, films and bags [39]. Sedničková et al. [40] demonstrated that PHB is more susceptible to degradation than PLA. During composting, the amorphous parts of the polymer decompose faster than the crystalline parts, with PHB degrading at a faster rate than PLA. Bonnenfant et al. [41] examined the possibility of reusing PHBV-based materials (pure and loaded with 1% quercetin) as food packaging, investigating their structural evolution over different cycles (full cycle of reuse (food contact/detergent washing/food contact), and successive washing operations in water, detergent and NaOH aqueous solutions). It has been shown that quercetin (a natural polyphenol) can be used as a thermal stabilizer of PHBV during processing. The PHBV material was able to withstand multiple washing cycles in water and detergent (up to 20) without significant changes in its structure and properties, making it suitable for food packaging applications.

### 2.3. Starch-Based Polymers

Starch is one of the oldest and most abundant raw materials available, making it an ideal candidate for bio-based packaging. It is composed of two main components: amylose and amylopectin. Amylose is a linear polymer made up of glucose units connected by α-(1,4) glycosidic bonds, while amylopectin, the predominant component, features a branched structure, with a linear backbone of α-(1,4)-linked glucose units and side branches formed via α-(1,6) glycosidic bonds [42]. Starch-based packaging is a sustainable material, offering a biodegradable and renewable alternative to conventional petroleum-based plastics. It is attractive for food packaging because it is abundant, inexpensive, biodegradable, compostable, and non-toxic, making it safe for direct contact with food products. Starches rich in amylose provide greater strength, hardness, and crystallization, while those rich in amylopectin enhance flexibility [43]. They are found in abundance in many agricultural crops and can be processed into bioplastics either alone or in combination with other biodegradable polymers [44]. It has been reported that starch can be processed by extrusion, which involves swelling of the starch, loss of birefringence, melting and starch granules solubilization [45]. Several studies have shown that starch extraction is a key step in the production of biodegradable starch-based films for food packaging [46,47]. De Paola et al. [48] developed potato starch-based films, using different fractions of plasticizer (glycerol), for the generation of biodegradable food packaging. The glycerol/starch ratios (*w*/*w*) were selected at 0.5, 0.6 and 0.7, respectively. The results showed that samples prepared with 10% starch and glycerol content between 5 and 6% are the most suitable film-forming suspensions for tape casting. Mileti et al. [49] reported the preparation of starch-based food films enriched with anthocyanins extracts. It has been demonstrated that the anthocyanin-starch-glycerol system may be suitable for the preparation of active packaging films and smart packaging films, as anthocyanins extracted from food industry waste exhibit antioxidant effect and structuring capacity in starch-based packaging films.

**Figure 4:**
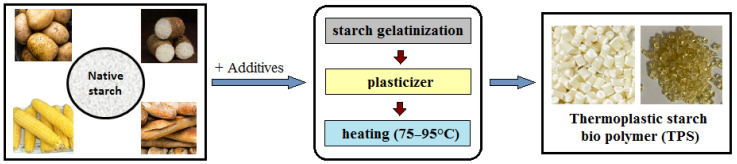
Schematic process for TPS using raw starch.

Starch, derived from renewable natural sources such as corn, potatoes, or cassava, becomes thermoplastic when heated with plasticizers (e.g., glycerol (GLY) or sorbitol) to a temperature above the starch’s gelatinization temperature (typically 75–95 °C), forming thermoplastic starch (TPS) which is a moldable, film-forming material that can be used in packaging (see Figure 4) [50,51,52,53,54] (Figure 4). The relationship between starch composition and TPS properties does not fully capture the complexities encountered in practical applications, where factors such as plasticizer interactions, environmental conditions (e.g., humidity), and polymer chain mobility play a critical role in determining material performance, highlighting the need to customize starch formulations for targeted applications [55].

### 2.4. Cellulose-Based Polymers

Cellulose is the most abundant organic polymer on Earth, extracted from wood (hardwood and softwood), cotton, and other plant fibers (e.g., straw and husks). It is a natural linear polymer composed of β-(1,4)-linked D-glucose units [56]. It plays a vital role in the development sustainable, functional, and safe food packaging. Cellulose naturally degrades in the environment, making it a more ecological option for packaging that minimizes pollution in the long term. Being a natural material, cellulose is safe for direct contact with food and does not release harmful chemicals, making it compliant with food safety regulations.

Cellulose can function as a food filler or stabilizer, enhancing the texture, stability, and sensory qualities of food products [57]. It has been reported that cellulose can be extracted from plant biomass by pre-hydrolysis, pulping and bleaching to obtain cellulose fiber. This cellulose fiber is a renewable and green resource of bioactive polymer material, supporting a circular economy [58]. Some previous studies have shown that cellulose can be converted into cellophane, a transparent, biodegradable film widely used in packaging [59,60,61].

**Figure 5:**
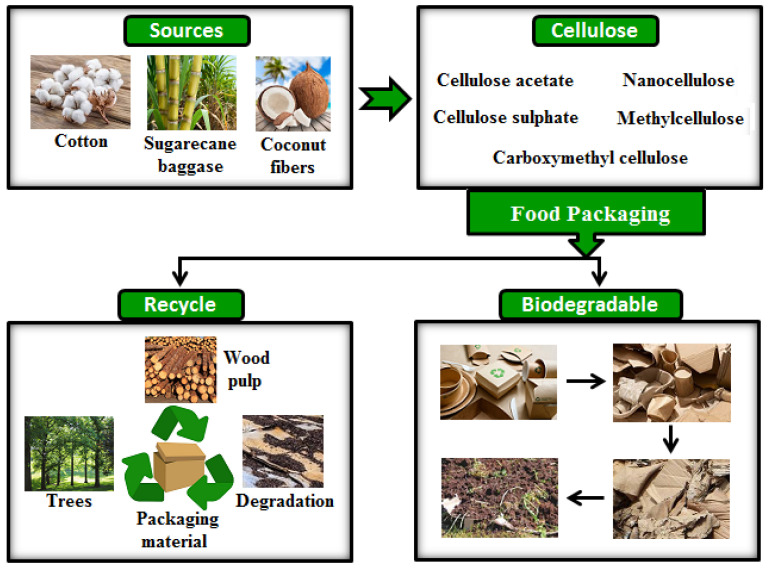
Common sources of cellulose derivatives used to develop biodegradable food packaging materials.

Native cellulose is rigid and insoluble in water and most organic solvents, but through chemical modification, it can be transformed into a variety of functional and processable cellulose derivatives with specific properties (e.g., water solubility, film-forming ability, mechanical strength, and thermal stability) for food packaging industries. Literature data have shown that cellulose derivatives, such as cellulose acetate (CA), cellulose sulphate (CS), carboxymethyl cellulose (CMC), nanocellulose (NC), and methylcellulose (MC), are being used to develop biodegradable food packaging materials [62,63,64,65,66]. They decompose naturally into harmless by-products, such as carbon dioxide, water, and biomass. Figure 5 illustrates the typical sources of cellulose and its derivatives, along with their application in food packaging to enhance the preservation of different food products.

It has been revealed that cellulose acetate (CA) can be produced by chemically modifying cellulose through acetylation, in which the hydroxyl groups in cellulose molecules are replaced with acetyl groups [67]. The result is a thermoplastic material that maintains many of the natural advantages of cellulose while achieving improved flexibility, durability, and resistance to moisture [68,69]. Cellulose acetate is commonly produced from renewable sources, such as wood pulp or cotton linters, and can be used for industrial processing and for end use in packaging. It plays a significant role in food packaging due to its favorable properties, such as film formability, biodegradability, low pollution profile, and relatively low cost.

Cellulose sulphate (CS) is a water-dispersible derivative of cellulose in which the hydroxyl groups (-OH) on the cellulose chain are replaced with sulfate groups (-OSO_3_H). This chemical modification (sulfonation) improves several of the properties of cellulose, including solubility, film-forming ability, and interaction with other materials [70,71]. Cellulose sulphate is a component in films and coatings that enhance barrier properties and improve food preservation.

Carboxymethyl cellulose (CMC) is a water-soluble cellulose derivative (eco-friendly material) produced by chemically modifying cellulose to introduce carboxymethyl groups (-CH_2_-COOH). It occurs as a white to yellowish-white powder and is known for its viscosity, and film-forming ability. Due to its biocompatibility and non-toxic nature, CMC plays a role in enhancing the shelf-life, quality and sustainability of packaged food products. [72,73,74]. Hussain et al. [75] reported the preparation of biodegradable films based on biopolymers (carboxymethyl cellulose (CMC), poly(vinyl alcohol) (PVA), and ascorbic acid (AA)), which can be used as a promising candidates in the food packaging industry. Kong et al. [76] described the preparation methods and properties of biofilms made from carboxymethyl cellulose (CMC) and chitosan (CS), which can be utilized as food packaging materials.

Nanocellulose is a renewable nanomaterial derived from plant biomass, such as wood, agricultural residues, or bacterial fermentation, and is commonly used as a reinforcing agent in biodegradable films made from starch, PLA, gelatin, and other biopolymers. Its use in packaging helps reduce plastic waste and create compostable and recyclable packaging. It has been shown that the production of nanocellulose involves the decomposition of purified cellulose into nano-sized structures using physical, chemical, or biological methods. Depending on the source and preparation method, different types of nanocellulose can be obtained, such as cellulose nanofibers and cellulose nanocrystals (obtained chemically or physically by the “top-down” method), and bacterial cellulose (biosynthesized by the “bottom-up” method) [77].

Derived from plant-based cellulose (e.g., wood pulp, cotton fibers, or agricultural residues (straw, corn stalks, and sugarcane baggase)), methylcellulose is non-toxic, biodegradable and film-forming, making it an ideal material for environmentally friendly packaging solutions [78]. Methylcellulose (MC) is synthesized by chemically modifying cellulose with methyl groups (-CH_3_). This process involves the reaction of cellulose with a methylating agent (e.g., dimethyl sulfate (DMS) or methyl chloride) in the presence of an alkaline solution (e.g., sodium hydroxide). Replacing some of the hydroxyl (-OH) groups in cellulose with methyl groups improves the solubility and film-forming ability of cellulose, while maintaining its natural biodegradability [79]. Du et al. [80] reported the preparation of gelatin-based films, using hydroxypropyl methylcellulose (HPMC) and carboxymethyl cellulose (CMC), which can be used as food packaging materials.

## 3. Advantages of Bio-Based Polymers Used in Food Packaging

The production of bio-based polymers typically results in a lower carbon footprint compared to conventional plastics (e.g., polyethylene (PE), polypropylene (PP), polystyrene (PS), polyvinyl chloride (PVC), and polyethylene terephthalate (PET)) [81,82]. This is because they are derived from renewable resources (e.g., starch, sugars and vegetable oils) that absorb CO_2_ during their growth. Bio-based polymers, such as PLA, and PHB, can contribute to reducing greenhouse gas emissions associated with food packaging.

Table 1 indicates the main advantages of bio-based polymers (polylactic acid, polyhydroxyalkanoates, starch-based polymers, and cellulose-based polymers) used for food packaging applications.

The main advantage of PLA is its biodegradability. Unlike traditional plastics, which can persist in the environment for many years, PLA can break down into natural components, such as water and carbon dioxide, under industrial composting conditions. This characteristic helps reduce long-term environmental pollution and supports circular economy initiatives. It does not release harmful substances in contact with food. It has been reported that high-molecular-weight PLA is suitable for the manufacture of fibers, flexible films, non-woven materials, as well as rigid and durable materials [83]. PLA is compatible with various processing techniques, including injection molding, thermoforming, and extraction. This versatility allows manufacturers to create a wide range of packaging products with different shapes and properties [84,85]. PLA film offers good transparency, and barrier properties compared to some petroleum-based plastics (e.g., polyethylene terephthalate (PET) [86]. These characteristics help maintain the freshness and quality of food, while providing a visually attractive packaging solution.

PHAs exhibit good flexibility, tensile strength, and thermal stability, making them versatile for various packaging formats, including films, trays, and containers. PHAs are non-toxic and biocompatible, making them safe for direct food contact. Some studies have shown that mixing thermoplastic starch (TPS) with synthetic polymers, usually aliphatic, for example polylactide (PLA) or poly-ε-caprolactone (PCL), leads to an improvement in mechanical properties [87].

The main advantages of starch in food packaging include its eco-friendliness, renewability, and cost-effectiveness. From a cost perspective, starch holds a clear advantage due to its abundance as an agricultural by-product, often derived from food processing streams. As a result, it is significantly less expensive than biopolymers produced through fermentation or chemical synthesis (e.g., PLA or PHA). Its low price and broad availability make it an appealing option as a filler or base material in bioplastic production [88]. Under proper conditions, many starch-based polymers are industrially compostable, meaning they decompose into carbon dioxide, water, and biomass within a relatively short period. This characteristic aligns with global efforts to reduce environmental impact and support circular economy practices. Literature data has shown that the important advantage of starch-based films over other degradable polymers is their colorless and transparent nature, which ensures that the visual appearance of the package is not influenced by the food product. Starch-based smart packaging systems often incorporate indicators that provide clear, visual, and quantitative or semi-quantitative information about the condition of the packaged food. These include freshness indicators, which respond to specific gases produced during storage to reflect remaining shelf life, and time-temperature indicators, which track cumulative temperature exposure over time to estimate product freshness [89].

Cellulose exhibits excellent film-forming abilities, making it suitable for the production of transparent flexible films that can be used to wrap or cover food products. These films provide a barrier against oxygen and grease, which helps to extend the shelf life of various food items. Furthermore, cellulose is a safe and non-toxic material, which is crucial for direct contact with food. Modified forms of cellulose, such as cellulose acetate (CA) or nanocellulose, can provide good barriers against oxygen, oils, and aromas, critical features for preserving food quality and shelf life. Cellulose sulphate (CS) exhibits excellent film-forming properties, allowing it to create transparent, flexible, and uniform films that can be used to wrap or coat food products. This makes it suitable for primary food packaging where visual appearance and product visibility are important [90]. CS is non-toxic and biocompostable, making it safe for food contact applications when properly processed [91]. Carboxymethyl cellulose (CMC) can produce smooth, flexible and transparent films ideal for packaging. CMC provides good resistance against grease, making it suitable for fatty food packaging. It can be used as an edible film or coating to extend the shelf life of fresh produce [92]. Nanocellulose exhibits superior tensile strength, enhancing the durability of packaging films. It provides strong barriers to gases (e.g., oxygen), improving food shelf life. NC films are lightweight and can be nearly invisible, suitable for modern packaging needs. It can be used to strengthen other biopolymers matrices, such as PLA, CMS, or starch. Methylcellulose (MC) creates transparent, flexible films with good gas and oil barrier properties. MC can be mixed with other polymers to improve the physicochemical properties. Lino et al. [93] demonstrated that the methylcellulose films containing 70% nanoparticles (poly-ε-caprolactone nanocapsules) can be considered an innovative nanomaterial for food preservation, offering the potential to extend the shelf life of perishable food products. Irimia et al. [94] developed an innovative approach for producing cellulose-based active packaging by using gamma irradiation as an environmentally friendly activation method, combined with clove essential oil and cold-pressed rosehip seed oil as natural bioactive compounds. The final results indicated that the obtained materials can be utilized in the food industry as active packages.

Studies have shown that bacterial cellulose (BC) films offer superior transparency compared to other biodegradable materials (e.g., chitosan–polyvinyl alcohol films). Nonetheless, in some cases, opacity can be beneficial, as it helps block ultraviolet (UV) radiation, a key contributor to lipid oxidation in food products [95]. They reported the production of biodegradable polymer composites from cellulose fibers from empty oil palm fruit bunches (OPEFB, different concentrations) and polylactic acid (PLA). The results indicated that polymer composites with a higher composition of cellulose fibers of OPEFB degrade more easily in the environment [96]. 

## 4. Properties of Bio-Based Polymers

Bio-based polymers are gaining popularity due to their potential to reduce environmental impact and dependence on fossil fuels. Their properties vary depending on the source and manufacturing process, but they often offer a unique combination of biodegradability, functionality, and performance [97].

Literature data has shown that PLA exhibits favorable properties, including biodegradability, biocompatibility, and mechanical strength, making it an ideal candidate for eco-friendly packaging solutions [98,99,100]. PHAs are resistant to water and moisture and present a high range of in-use temperature and low oxygen and water vapor permeability [101,102,103]. The mechanical, thermal, and physical properties of PHAs depend on the monomer composition, microstructure, and molecular weight distribution [102,103]. Starch-based polymers are highly customizable in terms of strength, flexibility, and barrier properties, although they typically have lower moisture resistance compared to synthetic polymers. It was reported that the physicochemical properties of cellulose, such as rigidity, strength, and extractability, are influenced by the relative proportions of its crystalline and amorphous regions. The crystalline regions contribute to tensile strength and rigidity, while the amorphous regions provide flexibility and ductility [104]. Cellulose-based polymers offer excellent mechanical strength, transparency, and resistance to oils and fats, making them suitable for packaging a variety of food products (e.g., dry foods and snacks). Cellulose-based films have been reported to possess good tensile strength and flexibility, allowing them to protect foods during handling and transportation [105].

### 4.1. Mechanical Properties

The mechanical properties of bio-based polymers, such as tensile strength, elasticity, and impact resistance, are highly dependent on their chemical structure and processing method [106].

Literature data has shown that polylactic acid (PLA) exhibits good tensile strength and stiffness, comparable to those of PE, PP, PS and PET, making it suitable for rigid packaging applications [107,108,109,110,111]. PLA has been shown to exhibit different mechanical properties depending on the preparation method. Oksiuta et al. [109] indicated that the PLA, prepared by extrusion and compressed under the pressure of ~10 MPa, has a tensile strength of 50.1 MPa, an elongation at break of ~2%, and a Young’s modulus of ~3467 MPa. Domenek et al. [112] reported that PLA is a glassy polymer at room temperature, exhibiting a tensile strength of around 60 MPa, an elongation at break of ~5%, and a Young’s modulus in the range of 3–4 GPa.

Some research studies have demonstrated that the addition of polycaprolactone (PCL) to PLA gives PLA sufficient flexibility to be used in packaging for applications such as those requiring flexible materials adapted to different geometries (household items) or for the extrusion of films for food preservation [112,113]. As previously noted, PHA-based materials, such as PHB exhibits mechanical properties are similar to those of conventional non-biodegradable plastics frequently used, such as PP, PE, and PET [111,114,115,116,117,118]. It has been shown that, while more flexible than PHB, PHBV maintains good tensile strength and dimensional stability [119,120,121,122,123].

Starch-based polymers may be more brittle and less durable unless blended with plasticizers or other polymers. Several researchers have shown that plasticizer concentration significantly influences the mechanical properties of starch-based films; specifically, higher plasticizer levels tend to reduce Young’s modulus and tensile strength, while enhancing the film’s elongation [124]. Starch can be combined with other bio-based materials (e.g., poly(lactic acid), polyhydroxyalkanoates (PHA), polycaprolactone (PCL), or polybutylene adipate terephthalate (PBAT)) or modified with various additives (e.g., glycerol (GLY)) to enhance the moisture resistance and mechanical strength. It has been shown that the use of cellulose nanofibers (CNFs) [125] or cellulose nanocrystals s (CNCs) [126] can improve the mechanical properties of starch-based thermoplastic (TPS) films. Almeida et al. [127] demonstrated that the mechanical properties of nanocomposite films of thermoplastic starch (TPS) can be enhanced by addition of bacterial nanocellulose (BNC, ≥5% *w*/*w*) and of gallic acid (GA, 1 and 1.5% *w*/*w*). It was found that TPS reinforced with BNC and GA exhibits a Young’s modulus between 1.2–2.0 GPa and a tensile strength between 23–39 MPa, compared to TPS which has a Young’s modulus of 1.0 GPa and a tensile strength of 20 MPa. These results showed that TPS-BNC-GA nanocomposites can be used as sustainable and eco-friendly film materials for active food packaging. Dominic et al. [128] reported the preparation of green thermoplastic starch (TPS) nanocomposite films using cellulose nanofibers (CNFs) in different concentrations (0, 1, 2, 3, and 4 wt%). The results indicated that the TPS/CNF green nanocomposites containing 3 wt% CNFs have remarkable tensile strength (~161%), and tensile modulus (~167%), and can be used as alternatives for sustainable packaging. Mileti et al. [129] studied the mechanical properties of starch films loaded with tannin used as an active additive). The tannin concentration used in the study was investigated in the range of 0–3% (*w*/*w*). The results indicated that the plastic and deformable films (elastic modulus = 1.96 MPa and elongational at break = 189%) can be obtained at low tannin fractions, while at a higher concentration, stiffer films (elastic modulus = 12 MPa and elongational at break = 10%) can be realized, with enhanced hydrophobic properties. The tannin concentration of 1.7% (*w*/*w*) demonstrated the highest potential for industrial purposes. Gurunathan et al. [130] developed a starch-based bioplastics by combining biopolymers (derived from corn and potato) with calcium carbonate as a filler (quantities of 2 g, 2.5 g, and 3 g), and glycerol-sorbitol as plasticizers. The bioplastic containing 3 g of calcium carbonate demonstrated an improved tensile strength of 6.08 MPa, making it suitable for rigid packaging and other uses such as food containers and shopping bags.

Although cellulose is used as a biodegradable material, it has low mechanical strength and has opacity [131]. In previous papers, it was shown that the mechanical properties (e.g., tensile strength and elongation) of cellulose-based films can be enhanced by incorporation of fillers into polymer matrix [132,133]. Hussain et al. [134] reported the preparation of biodegradable films by combining hemicellulose B (HB) with methylcellulose (MC) and carboxymethyl cellulose (CMC) at different mass ratios (HB:MC/CMC ratios of 80:20, 70:30, and 60:40). The mechanical properties (tensile stress ~10.45 MP, elongation at break ~137.17%, elastic modulus, and toughness) of these films were improved by the addition of plasticizers, glycerol (GLY) and polyethylene glycol (PEG). Sridhara et al. [135] revealed that the mechanical properties (elastic modulus and the flexural properties) of cellulose-based composites can be influenced by the cellulose fiber content (5, 10, 15, 20, and 25%). The highest tensile strength of 66.17 MPa was obtained for a 20% composition, followed by 64.76 MPa for a 25% composition for dry samples. The flexural modulus showed significant improvements (from 2.45 GPa to 3.84 GPa), and the flexural strength showed gradual improvements (from 105 MPa to 113.6 MPa) with increasing cellulose composition. Chen et al. [136] realized cellulose sulfate films using β-cyclodextrin (β -CD) and mustard essential oil (MO). It was demonstrated that the mechanical properties of films were significantly affected by the amount of MO incorporated into the composite structure. The films exhibited a tensile strength between 39.5 MPa and 46.3 MPa, an elongation at break between 47.3% and 21.3%, and an elastic modulus between 969 MPa and 1398 MPa. Vidal et al. [137] developed films made from carboxymethyl cellulose (CMC) enriched with green coffee oil (GCO) and extracts from green coffee residue, cake and sediments (in different proportions (20–40%)) for potential use in food packaging. The mechanical properties of films were evaluated and it was observed that the tensile strength of the films decreased significantly from 58 MPa to 3 MPa, while elongation increased from 28% to 156% (*p* < 0.05), as the concentration of extracts increased.

The general mechanical properties of PLA, PHAs, starch-based polymers, and cellulose-based polymers, commonly used for food packaging, are given in Table 2.

**Figure 6:**
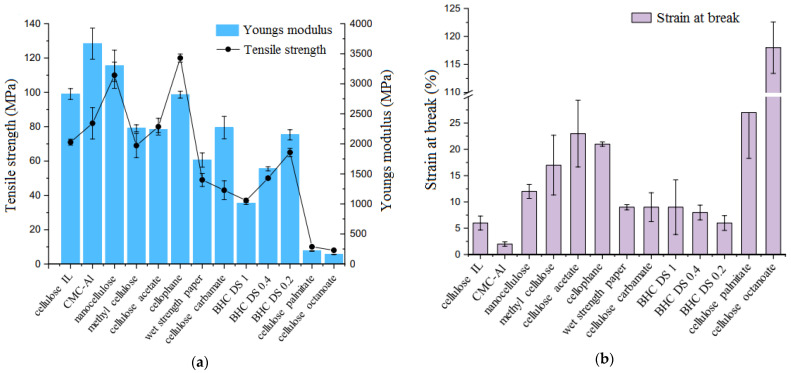
Mechanical properties of cellulose-based films: (**a**) Tensile strength (MPa) and Young’s modulus (MPa); (**b**) Percentage strain at break (%) (copyright, reproduced with permission from ref. [138]).

Leppänen et al. [138] studied different type of cellulose-based films and showed how chemical substitution and the substituent itself affect the mechanical behavior of cellulose materials. The materials tested in this study were cellulose regenerated from ionic liquid (cellulose IL), carboxymethyl cellulose (CMC) crosslinked by aluminum salt (Al-salt), nanocellulose, methyl cellulose, cellulose acetate, cellophane, wet strength paper, cellulose carbamate, butylated hemicellulose: DS: 1, DS: 0.4, and DS: 0.2, cellulose palmitate, and cellulose octanoate. The results showed that cellophane film has the highest tensile strength (120 MPa) with Young’s modulus of 2820 MPa, nanocellulose having the second highest values with tensile strength of 110 MPa and Young’s modulus of 3300 MPa. Cellulose octanoate film showed clearly the highest percentage strain at break (118%) (see Figure 6). It was also observed that differences in the degree of substitution have a smaller effect on the mechanical properties (tensile strength, Young’s modulus, and percentage strain at break) than the side chain length.

### 4.2. Barrier Properties

The barrier properties of biopolymers, such as resistance to oxygen (O_2_), moisture, carbon dioxide (CO_2_), light, and contaminants, are crucial in food packaging to separate the food product from the external environment [139,140].

Bio-based polymers are generally hydrophilic (e.g., nanocellulose, cellulose sulphate), increasing humidity increases gas permeability. To manage or control the hydrophilic nature of bio-based materials, they can be coated externally with water-resistant materials, cross-linked with inorganic fillers, or blended with other water-resistant materials [140].

Literature data showed that PLA have a moderate gas barrier performance. Compared to conventional plastics (e.g., polyethylene (PE) or polystyrene (PS), PLA performs better in preventing oxygen permeation. The oxygen transmission rate for PLA is ~400–600 cc/m^2^/day at 23 °C (varies by grade and thickness of film). PLA has high water vapor transmission (WVTR, ~20–40 g/m^2^/day at 38 °C), making it less ideal for products that require protection against humidity or dehydration, such as baked goods or dairy [141,142,143]. It has been reported that the barrier performance of PLA can be improved by blending PLA with other polymers (e.g., PHA or polycaprolactone), combining PLA with high barrier materials, incorporating filler nanomaterials (e.g., cellulose nanocrystals) into PLA for reduced permeability [144]. Several authors have studied the gaseous transfer of O_2_, CO_2_, and H_2_O through PLA films, examining how factors, such as additives, and film structure influence permeability [145,146,147,148]. Stoll et al. [149] examined the incorporation of acetyl tri-butyl citrate (ATBC) as a plasticizer in PLA films and found that it resulted in a reduction in the oxygen barrier performance of the films. The films blocked up to 95% of UVA transmission and 90% of UVB transmission. Mulla et al. [150] reported the barrier properties of PLA-based nanocomposites prepared by reinforcing different nanoparticles. Studies have shown that the obtained PLA-based nanocomposites exhibit a decrease in water vapor and oxygen permeability compared to pure PLA films. Shankar et al. [151] demonstrated that the water vapor barrier (WVP) of the PLA films blending with zinc oxide nanoparticles (ZnO NPs) decreased significantly by 30.5% (WVP of 2.16 × 10^−11^ g m/m^2^·Pa·s), compared to the bare PLA film (WVP of 3.11 × 10^−11^ g m/m^2^·Pa·s).

Certain PHAs provide good resistance to oxygen and moisture, which helps preserve the shelf life and quality of packaged food. This makes them suitable for dry foods, snacks, and bakery items [152,153,154]. Literature data has shown that PHA represents only 1.4% of the biopolymer market, despite its good barrier properties (superior compared to PLA and similar to PET), but its production levels are expected to increase [155]. Papadopoulou et al. [156] prepared composites of poly(3-hydroxybutyrate) (PHB) with graphene nanoplatelets (GnP) at various concentrations and investigated the oxygen permeability behavior. The results showed that the oxygen permeability reduction reaches 98% for the sample with 15% GnP (hot-pressed sample), indicating that this sample is a very promising candidate for packaging applications.

To improve the functionality of starch-based packaging, current research has focused on the use of nanomaterials (e.g., nanoclays or cellulose nanofibers) to enhance barrier properties. In addition, blending starch with different polymers (e.g., polyethylene) can improve the barrier qualities of packaging material [157]. Wang et al. [158] demonstrated the barrier properties of starch film prepared using dialdehyde starch (DAS) as the matrix, polyvinyl alcohol (PVA) as a toughening agent, and cellulose nanofibrils (CNF) as a reinforcing agent. The final results indicated that the composite film exhibits superior fruit preservation ability compared to polyethylene packaging film and can be utilized as a biobased high strength barrier film.

On the other hand, cellulose-based materials can exhibit excellent oxygen barrier properties, which can be improved through multilayer structures or coatings. Cellulose sulphate-based films can provide moderate barriers to gases and oils, helping to reduce oxidation and moisture migration in packaged foods. Zhao et al. [159] reported the preparation of hemicellulose film, using sodium trimetaphosphate (STMP) as esterification agent. The barrier performance of the modified hemicellulose film was evaluated and verified by an apple preservation test, in which changes such as water loss and discoloration were visually observed over time. Preliminary results showed a good prospect for the prepared hemicellulose film in the food packaging industry.

### 4.3. Antimicrobial Property

Bio-based polymers with antimicrobial property are extensively used in active food packaging to inhibit spoilage microorganisms, thereby extending the shelf life of perishable products. Antimicrobial biopolymers are used for biodegradable seed coatings or films that protect crops from pathogenic microorganisms during germination and early growth [160].

It have been reported that the PLA does not exhibit intrinsic resistance to microbial contamination, making it susceptible to colonization by bacteria and fungi in certain environments. Various strategies have been used to create antimicrobial PLA-based materials, including: incorporating essential oils (e.g., oregano, thyme) into PLA films by casting or solvent extraction, mixing plant extracts (e.g., green tea, rosemary) with PLA to inhibit bacterial growth, using inorganic nanoparticles (e.g., silver nanoparticles (Ag NPs), zinc oxide (ZnO), and titanium dioxide (TiO_2_)). Literature data has shown that antimicrobial PLA films help prevent microbial spoilage, increasing the safety and shelf life of perishable products (e.g., fruits, meat, and dairy). Active packaging using PLA blended with essential oils or nanoparticles has demonstrated potent inhibition of *Escherichia coli*, *Listeria monocytogenes*, and *Staphylococcus aureus* [161]. Dejene et al. [162] investigated the development of a biocomposite packaging material by reinforcing PLA with Enset fibers (EFs) and zinc oxide nanoparticles (ZnO NPs), aiming to improve the preservation a traditional Ethiopian dish. Various formulations were prepared by incorporating different concentrations of EFs (5%, 15%, and 25%) and ZnO NPs (0%, 5%, and 10%) into the PLA matrix. The optimal blend, containing 6% EFs and 6.7% ZnO NPs, demonstrated strong antifungal activity, with inhibition zones measuring 1.5 cm, 1.47 cm, and 1.41 cm against *Aspergillus niger*, *Penicillium*, and *Rhizopus*, respectively. The shelf life of Ethiopian dish was extended to more than 8 days, a significant improvement over the usual 2–3 days, highlighting the material’s potential to reduce spoilage and improve food safety.

PHAs serve as an excellent matrix for developing antimicrobial systems due to their non-toxic nature, film-forming ability, and compatibility with bioactive compounds [163,164]. The hydrophobic nature of PHA can also contribute to reduced microbial adhesion in some cases, but this effect alone is typically insufficient for food preservation. Various additives (e.g., essential oils, polyphenolic-rich plant extracts, and nanoparticles) have been used to improve the antimicrobial activity of PHA [165]. It have been reported that the nanoparticles, such as Ag NPs and ZnO, can generate reactive oxygen species leading to cell death [166]. Ibrahim et al. [167] reported the preparation of bionanocomposite based on PHBV reinforced with Ag–ZnO nanoparticles at concentration of 1%, 3%, 5% and 10%. The results indicated that the bionanocomposite PHBV/Ag–ZnO (10%) was the most potent against *S. aureus* and *E. coli*, respectively.

Cellulose can be combined with natural antimicrobial agents, such as essential oils or plant extracts, to create active packaging systems that inhibit microbial growth and improve food safety. Due to the changed sulfate groups, cellulose sulfate can interact with certain antimicrobial agents or nanoparticles, making it suitable for developing active food packaging. Such packaging can help inhibit microbial growth, thereby extending shelf life and improving food safety. Ballesteros et al. [168] developed coatings based on carboxymethyl cellulose (CMC) enriched with bioactive compounds extracted from spent coffee grounds (SCG) to extend the shelf life of fresh goldenberries. The microbiological quality of the fruit was monitored over 12 and 28 days of storage at 20 °C with 65% relative humidity, and at 4 °C with 95% relative humidity, respectively. The best results were observed for the CMC-based coating containing 0.20% (*w*/*v*) of the polysaccharide-rich extract, successfully inhibiting the growth of fungal species (*Alternaria* sp., *Phoma violacea*, *Penicillium expansum*, *Cladosporium cladosporioides*, *Fusarium culmorum*, and *Botrytis cinerea*). Yang et al. [169] prepared composite films based on carboxymethyl cellulose (CMC) by incorporating the polyphenol-rich extract derived from coffee husk (CHE) along with carbon dot (CDs, various contents of 0, 1, 3, 5, and 7%, *w*/*w*). The results showed that the composite films exhibit antibacterial capabilities against *L. monocytogenes* and *E. coli*. Santos et al. [170] studied the antimicrobial activity of cellulose acetate films incorporated with essential oils of cinnamon, sweet fennel, and oregano (natural antimicrobial agents) against the bacteria *E. coli* and *S. aureus*, as well as the fungus *Penicillium* spp. The results indicated that the films into oregano and the film with combination cinnamon + oregano presented the highest antimicrobial activity against bacteria (inhibition zones of 0.99 cm (*E. coli*) and 3.75 cm (*S. aureus*)), and fungus (inhibition zone of 2.67 cm) tested.

### 4.4. Eco-Friendly Behavior

PLA and chitosan have attracted interest due to their environmentally friendly properties, demonstrating potential for use in food packaging applications. Advances in the field of biodegradable packaging polymers highlight a growing trend towards sustainable alternatives that aim to address environmental challenges while ensuring proper food preservation [171]. PLA production typically generates fewer greenhouse gas emissions than conventional plastics. Because PLA is derived from renewable biomass, the carbon dioxide absorbed by plants during growth partially offsets the emissions from its production and disposal, making PLA a more environmentally friendly option. Moldovan et al. developed PLA-based composites embedded with grape pomace or copper particles, improving sustainability by recycling agricultural waste and offering both eco-friendly and functional properties, positioning the material as a promising candidate for active food packaging [172].

PHAs represent an important step towards a circular and environmentally friendly packaging industry due to their biodegradable nature, food safety compatibility and versatile material properties [173].

Starch, a natural polysaccharide found in plants, such as corn, potatoes, and cassava, plays an increasingly important role in the development of eco-friendly food packaging [174]. Although biodegradable, many starch-based materials require specific composting conditions, such as temperature, humidity, and microbial activity, to degrade efficiently. In areas without industrial composting infrastructure, these materials may not break down effectively, reducing their environmental benefits.

Derived from plant cell walls, cellulose offers a renewable and eco-friendly solution for food packaging, combining functionality with sustainability. Cellulose acetate (CA) is considered a bio-based material because it is derived from renewable biomass, contributing to reduced dependency on fossil resources [175]. CA contributes to environmental sustainability because it is non-toxic and safe for food contact and biomedical use. Nazari et al. [176] reported the preparation of nanostructures by incorporation of Ziziphora clinopodioides essential oils (ZEO)-loaded chitosan nanoparticles into cellulose acetate (CA) nanofibers. The findings highlighted the promising effectiveness of novel nanostructures in active packaging for preserving the quality of perishable food products. Carboxymethyl cellulose (CMC) can be used to create biodegradable films that replace conventional plastics in wrapping and bags. Its use in packaging can help minimize environmental impact and supports the development of compostable materials. Cellulose sulfate (CS) is biodegradable and non-toxic, providing an environmentally friendly alternative to petroleum-based plastics, and helping reduce long-term environment pollution. Through its use in food packaging, it supports the global effort to reduce plastic waste and adopt more sustainable materials. Methylcellulose (MC) decomposes naturally without leaving harmful residues, aligning with environmental goals of reducing plastic waste. MC can be used alone or blended with other biopolymers (e.g., starch, or gelatin) to produce compostable packaging films.

## 5. Applications of Bio-Based Polymers

**Figure 7:**
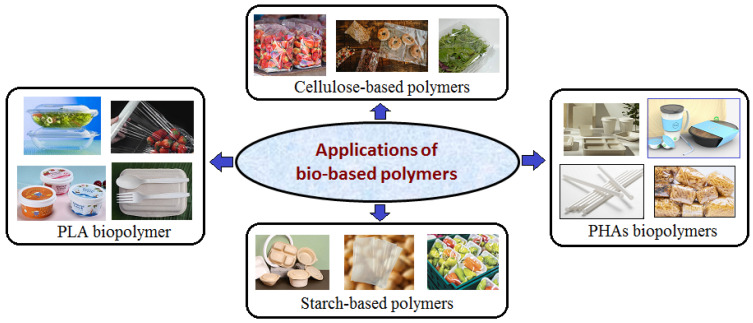
Schematic illustration of PLA, PHAs, starch-based polymers, and cellulose-based polymers used for various food packaging applications.

Figure 7 illustrates the primary applications of bio-based polymers in the food packaging industry.

PLA-based materials are widely used for various food packaging applications, including: (1) containers and trays (for fresh produce, ready-to-eat meals, and bakery products), (2) bottles and cups (used for cold beverages and dairy products), and (3) films and wraps (employed as cling films or as part of multilayer barrier films for snacks and perishables). These products benefit from PLA’s clarity, stiffness, and printability, making them both functional and visually appealing. PLA is widely used for items such as clear food containers, cups and lids, and food trays [177,178].

Due to their excellent biodegradability, non-toxicity, and compatibility with food contact standards, PHAs are increasingly being used in the food packaging industry for products, such as (1) rigid containers and trays (ideal for fruits, vegetables, snacks, and takeaway meals), (2) films and wraps (used for wrapping fresh produce, bakery goods, and deli items), and (3) coatings and laminates (applied to paper-based packaging to improve moisture and gas barriers) [179,180].

Starch can be used alone or in combination with other materials to create different types of packaging. One of most common applications is the production of biodegradable films and bags. These films can be used to package fruits, vegetables, baked goods, and other perishable products. Starch is also used in foam packaging materials, often as an alternative to polystyrene foam. These foams are lightweight, protective, and ideal for protecting fragile foods during transport. Additionally, edible coatings made from starch have been explored to extend the shelf life of fresh produce by acting as a barrier against oxygen and moisture [181]. Starch-based polymers are used in a variety of food packaging formats, such as (1) flexible films and wraps (for packaging fruits, vegetables, dry snacks, and bakery products), (2) trays, containers, and cutlery (often used for takeaway and fast-food services), and (3) coatings (applied to paper or cardboard to improve moisture and grease resistance) [182,183,184].

Cellulose can be applied as a coating to paper or cardboard to improve barrier properties and water resistance. Cellulose-based polymers are used in both rigid and flexible food packaging formats, including: (1) films and wraps (used for packaging fruits, vegetables, baked goods, and confectionery), (2) coatings (applied to paper or cardboard to improve grease, moisture, and oxygen resistance), and (3) composites (blended with other biopolymers, such as PLA or starch, to create stronger and more functional packaging materials) [185,186].

## 6. Disadvantages of Bio-Based Polymers Used in Food Packaging

Currently, bio-based polymers are more expensive to produce than conventional plastics due to costly feedstocks, less mature production technologies, and limited economies of scale. This makes them less competitive in price-sensitive markets and restricts large-scale adoption. Although improving, many bio-based polymers still lack some of the properties needed for high-performance food packaging, such as poor moisture resistance, lower mechanical strength or durability, and shorter shelf life for packaged products.

It has been reported that the recovery of PHB from biomass through solvent pre-treatment method has raised environmental concerns, as the high solvent usage not only impacts sustainability but also significantly increases the overall extraction cost [187]. PHAs can be sensitive to moisture and UV light, which may affect their stability and performance during storage. Proper packaging design and additives are often needed to mitigate these issues [188].

Research indicates that the primary limitation of bacterial cellulose in industrial applications is its high production cost, largely influenced by factors such as the composition of the growth medium, culture conditions, and the specific bacterial strains employed [189]. Cellulose sulphate (CS) exhibits a hydrophilic nature, meaning it readily absorbs water. This limits its use in packaging high-moisture foods unless combined with hydrophobic coatings or blended with other materials. Films made from pure CS may exhibit low mechanical strength and limited flexibility, making them prone to tearing or deformation. The durability and performance of these films can be achieved by reinforcing or modifying them with other biopolymers. CS does not perform well at high temperatures, which limits its use in applications involving heat, such as microwaveable or hot food packaging. It can degrade or lose structural integrity when exposed to heat. Compared to PLA and starch-based materials, CS is less commercially available. Its production is still relatively niche, which may lead to higher costs and limited scalability for industrial food packaging. Being hydrophilic, nanocellulose (NC), methylcellulose (MC), and carboxymethyl cellulose (CMC) absorbs moisture easily, limiting its use in high-humidity environments. Films made from CMC or MC may require plasticizers or blending with other biopolymers to improve strength.

It was reported that the starch presents several drawbacks as a packaging material, including its strong affinity for water, high sensitivity to environmental conditions, especially moisture, brittleness, and poor compatibility with hydrophobic synthetic polymers [190,191]. Poor water resistance limits the use of starch-based polymers for packaging wet or high-moisture food products unless blended with other materials or coated with protective layers. Pure starch-based films often have low mechanical strength, and poor flexibility, making them less durable compared to traditional plastics. Additives or blending with other biopolymers (e.g., PLA or PCL) are usually required to improve their performance. Because of their sensitivity to moisture and microbial degradation, starch-based materials can have a shorter shelf life, particularly in humid environments. This can lead to premature deterioration and compromise the protective function of the packaging. While starch itself is inexpensive, the processing and modification required to make starch-based polymers suitable for packaging can increase costs. Blending, plasticization, and the additions of functional additions make these materials more expensive than some traditional plastics [192].

Compared to conventional plastics, PLA (polylactic acid) has limited heat resistance, with a recommended maximum usage temperature of approximately 43 °C. Traditional materials, such as polypropylene, can tolerate much higher temperatures, up to around 104 °C. Consequently, PLA is not suitable for packaging hot liquids or foods (at high temperature between 70 °C and 95 °C), and its use is discouraged for items such as hot soups or beverages [193]. To overcome these limitations, bio-based polymers are often blended with other materials, which can complicate recycling or biodegradation. Compared to traditional plastics (e.g., polyethylene (PE) or polypropylene (PP)), PLA tends to be more brittle and has lower impact strength. This can lead to cracking or breaking under pressure or during transport. To overcome this, PLA is often blended with other polymers or plasticizers, which may compromise its compostability or increase costs. Although PLA is marketed as biodegradable and compostable, it requires specific industrial composting conditions (above 58 °C with controlled humidity and microbial activity) to break down efficiently. The cost of fermentation, polymerization, and post-processing makes PLA less economically attractive, especially for manufacturers in price-sensitive markets.

Most bio-based polymers are derived from food crops (e.g., corn and sugarcane), raising concerns about competition with food production, land use pressure, and potential increase in food prices. To address this, alternative sources such as agricultural waste, algae, and non-edible biomass are being explored.

Table 3 shows the main disadvantages of bio-based polymers (polylactic acid, polyhydroxyalkanoates, starch-based polymers, and cellulose-based polymers) used for food packaging applications.

## 7. Current Challenges and Future Research Directions

While bio-based polymers present a promising alternative to traditional plastics in food packaging, there are several challenges that need to be addressed:

➢ Cost

Currently, bio-based polymers are often more expensive to produce than petroleum-based plastics. This is mainly due to the higher cost of raw materials and production processes. However, as demand for biodegradable packaging increases and production expands, costs are expected to decrease over time. Processing and chemical modification of cellulose can increase costs compared to conventional plastic packaging.

Efforts are underway to develop eco-friendly and energy-efficient manufacturing processes, such as enzymatic modification, microwave-assisted synthesis, and solvent-free methods. These technologies can help reduce the environmental footprint and overall cost of bio-based polymers.

➢ End-of-life Considerations

While many bio-based polymers are biodegradable or compostable, their breakdown often requires specific conditions (e.g., industrial composting facilities). Without proper disposal infrastructure, these materials may not fully degrade, leading to waste accumulation in landfills or natural environments. Additionally, there is a lack of standardized regulations regarding the disposal and recycling of bio-based polymers.

Research is exploring the design of polymers that degrade efficiently not only in industrial composters, but also in home composting conditions or natural environments (soil, water). This would significantly expand the practical benefits of biodegradable packaging.

➢ Resource Competition

The production of bio-based polymers often relies on agricultural crops, such as corn and sugarcane. This raises concerns about land-use competition between food production and the fabrication of biomaterials. To mitigate this, researchers are exploring alternative feedstocks, such as algae, waste biomass, and non-food crops, to reduce the strain on food resources. This supports waste valorization in a circular economy.

To enhance performance of bio-based polymers for food packaging applications, future research is focusing on blending bio-based polymers with other natural or synthetic materials, or incorporating nanomaterials (e.g., nanocellulose, or nanoparticles). These approaches aim to improve barrier properties, strength, and antimicrobial activity without compromising biodegradability.

Research is ongoing to develop smart PLA materials that respond to microbial contamination by releasing active agents only when needed. There is also growing interest in using waste-derived antimicrobial extracts (e.g., from coffee grounds or fruit peels) for sustainable functionalization. With continued research, technological advancements, and improved infrastructures, PHAs have the potential to play a major role in the future of sustainable food packaging.

Research and innovation must continue to improve the water resistance and mechanical properties of cellulose for food packaging, by blending cellulose with other biodegradable polymers and developing functionalized coatings. As technology advances and consumer demand for sustainable products increases, cellulose is expected to play a key role in the future of environmentally responsible packaging.

As research continues, cellulose-based packaging is expected to evolve in the following directions: (i) development of multifunctional films with antimicrobial, antioxidant and smart characteristics; (ii) integration into active and intelligent packaging systems; (iii) use of cellulose derived from ecological synthesis and waste to reduce costs and improve sustainability and (iv) commercial expansion for mass production of biodegradable food packaging.

Ongoing research is aimed at improving the properties and expanding the applications of starch, which could lead to a significant reduction in plastic waste. With continued innovation and investment, starch-based packaging could become a leading solution in the global effort towards sustainability.

The growing demand for sustainable food packaging solutions is driving innovation in the development of bio-based polymers. Ongoing research into new feedstocks, production methods, and polymer blends aims to overcome the current limitations and make bio-based packaging more affordable, durable, and widely available.

As consumer preferences continue to shift toward more sustainable products, bio-based polymers are poised to play a key role in the transformation of food packaging, providing a more eco-friendly alternative to traditional plastics. However, achieving widespread adoption will require collaboration between researchers, manufacturers, policymakers, and consumers to ensure that bio-based polymers are produced, used, and disposed of in a way that maximizes their environmental benefits.

## 8. Conclusions

Bio-based polymers represent a promising innovation in the food packaging industry, offering an alternative to petroleum-based plastics with a potentially lower environmental impact. While challenges remain, ongoing advancements in material science and production technology suggest that bio-based polymers will play an increasingly important role in sustainable packaging solutions. By embracing these materials, the food industry can help pave the way toward a more sustainable future for both food packaging and the planet. Bio-based polymers, such as PLA, PHAs, starch-based polymers, and cellulose-based polymers, are already utilized in industrial production for food packaging applications.

To enhance the strength and heat resistance of PLA, PHAs, and starch-based nanocomposites, several strategies can be considered, such as: blending with other biopolymers (e.g., polycaprolactone (PCL), incorporating plasticizers to improve flexibility and reduce brittleness, and using filler nanomaterials (e.g., nanocellulose) to improve both mechanical and barrier properties.

It was concluded that PHAs have potential as matrices for active packaging systems where natural antimicrobial agents (e.g., essential oils or plant extracts) can be incorporated into the polymer matrix. Their biodegradability, bio-based origin, and safety make them excellent candidates for replacing conventional plastics in single-use and specialty applications. However, high costs, processing limitations, and inconsistent performance remain critical challenges.

Starch, derived from abundant and renewable sources (e.g., corn, potato, and cassava), is biodegradable, compostable, and non-toxic, making it a highly attractive material for eco-friendly packaging. Starch-based films can be used as carriers for antimicrobial agents (e.g., essential oils), pH indicators or spoilage sensors. Such active packaging systems can enhance food safety and reduce waste. To ensure better environmental performance, research is focused on making starch-based packaging fully compostable in the home setting and soil environments, avoiding the need for industrial composting facilities. Future research directions must focus on: material enhancements through blending, modification, and nanotechnology; functional packaging solutions; sustainable and cost-effective productions; and improved processing and end-of-life behavior.

Derived from renewable sources, such as wood, cotton, and agricultural residues, cellulose and its derivatives (cellulose acetate (CA), carboxymethyl cellulose (CMC), nanocellulose (NC), methylcellulose (MC), and cellulose sulphate (CS)) offer numerous advantages such as biodegradability, film-forming ability, transparency, and safety for food contact, making them ideal candidates for a wide range of food packaging applications. However, challenges such as moisture sensitivity, limited flexibility, processing complexity, and cost must be overcome. Future research directions should prioritize material modification for improved performance, eco-friendly production techniques, functional packaging solutions and scalable industrial integration.

While bio-based polymers still face challenges in terms of cost, performance in demanding applications, and scalability, ongoing research and innovation are steadily improving their properties. In the future, the integration of bio-based polymers into food packaging could help significantly reduce plastic waste, promote a circular economy, and minimize the environmental impact of the food packaging industry.

## Figures and Tables

**Table 1:** The main advantages of polylactic acid, polyhydroxyalkanoates, starch-based polymers, and cellulose-based polymers, used as bio-based polymers for food packaging.

Bio-Based Polymer	Advantages
Polylactic Acid (PLA)	Biodegradability and compostability; Renewable resource; Good printability; Non-toxic and safe for food contact; Reduction in greenhouse gas emissions; Compatibility with various processing techniques
Polyhydroxyalkanoates (PHAs)	Biodegradability in natural environments; Renewable resource origin; Biocompatibility and non-toxicity; Oxygen Barrier Properties.
Starch-based polymers	Degrade naturally in soil or compost; Low-cost raw material; Safe for direct contact with food and do not release harmful substances; Good film-forming ability.
Cellulose-based polymers	Decompose naturally without leaving toxic residues; Sustainable raw material that contributes to reducing dependence on fossil fuels; Cellulose films offer excellent barrier properties against oils, aromas, and gases; Non-toxic; Transparency and printability.

**Table 2:** The general mechanical properties of PLA, PHAs, starch-based polymers, and cellulose-based polymers, commonly used for food packaging.

Bio-Based Polymer	Tensile Strength (MPa)	Young’s Modulus (MPa)	Elongation at Break (%)	Refs.
PLA	50–62	3453–3750	1.2–7.8	[109,110,111]
PLA with PCL	46–55	2687–3555	18–65	[111,113]
PHA	14–40	3500–4622	0.4–2	[116,117]
PHB	20–45	800–2600	2–8	[118,119,120]
PHBV	2–21	1100–1300	30–44	[121,122,123]
TPS with CNFs	10–18	53–144	5.3–8.7	[125]
TPS with CNCs	1.7–2.4	28–33	77–81	[126]
TPS with BNC and gallic acid	23–39	1200–2000	3.4–4.1	[127]
Cellulose mixture with filler	38–52	989–1020	4.8–8	[132]
Cellulose pulp fibers with polyamide	48–66	4400–4600	3.5–6	[135]

**Table 3:** The main disadvantages of polylactic acid, polyhydroxyalkanoates, starch-based polymers, and cellulose-based polymers, used as bio-based polymers for food packaging.

Bio-Based Polymer	Disadvantages
Polylactic Acid (PLA)	Not suitable for hot-fill packaging (70–95 °C); May deform in hot environments, such as inside a car or microwave; Low water vapor and oxygen barrier resistance; Short shelf life for packaged food; More expensive than petroleum-based plastics.
Polyhydroxyalkanoates (PHAs)	High Production Costs; Thermal Instability; Sensitive to UV light; More sensitive to moisture and temperature.
Starch-based polymers	Poor moisture resistance; Mechanical weakness; Degrade or lose their properties over time under varying humidity and temperature conditions.
Cellulose-based polymers	Moisture Sensitivity; Processing and chemical modification of cellulose can increase costs.

## Data Availability

Data are contained within the article.
